# Ecology of phlebotomine sandflies and putative reservoir hosts of leishmaniasis in a border area in Northeastern Mexico: implications for the risk of transmission of *Leishmania mexicana* in Mexico and the USA

**DOI:** 10.1051/parasite/2017034

**Published:** 2017-08-21

**Authors:** Jorge J. Rodríguez-Rojas, Ángel Rodríguez-Moreno, Miriam Berzunza-Cruz, Gabriel Gutiérrez-Granados, Ingeborg Becker, Victor Sánchez-Cordero, Christopher R. Stephens, Ildefonso Fernández-Salas, Eduardo A. Rebollar-Téllez

**Affiliations:** 1 Laboratorio de Entomología Médica, Departamento de Zoología de Invertebrados, Facultad de Ciencias Biológicas, Universidad Autónoma de Nuevo León Av. Universidad S/N, Cd. Universitaria C.P. 66450 San Nicolás de los Garza Nuevo León México; 2 Departamento de Zoología Instituto de Biología, Universidad Nacional Autónoma de México, Circuito Exterior S/N C.P. 04510 Coyoacán Ciudad de México México; 3 Unidad de Investigación en Medicina Experimental, Facultad de Medicina, Universidad Nacional Autónoma de México, Dr. Balmis #148, Colonia Doctores C.P. 06726 Ciudad de México México; 4 Facultad de Estudios Superiores Zaragoza, Universidad Nacional Autónoma de México, Batalla 5 de mayo S/N esquina Fuerte de Loreto, Col. Ejército de Oriente Iztapalapa C.P. 09230 Ciudad de México México; 5 Centro de Ciencias de la Complejidad, Universidad Nacional Autónoma de México Circuito Exterior S/N. C.P. 04510 Cd. Universitaria, Ciudad de México México; 6 Instituto de Ciencias Nucleares, Universidad Nacional Autónoma de México Circuito Exterior S/N. C.P. 04510 Coyoacán Ciudad de México México; 7 Centro de Investigación en Ciencias de la Salud, Universidad Autónoma de Nuevo León Av. Carlos Canseco S/N. C.P. 64460 Mitras Centro, Monterrey Nuevo León México; 8 Centro Regional de Investigación en Salud Pública, Instituto Nacional de Salud Pública 19 Poniente Esquina 4ª Norte S/N. C.P. 30700 Centro Tapachula Chiapas México

## Abstract

Leishmaniases are a group of important diseases transmitted to humans through the bite of sandfly vectors. Several forms of leishmaniases are endemic in Mexico and especially in the Southeast region. In the Northeastern region, however, there have only been isolated reports of cases and scanty records of sandfly vectors. The main objective of this study was to analyze the diversity of sandflies and potential reservoir hosts of *Leishmania* spp. in the states of Nuevo León and Tamaulipas. Species richness and abundances of sandflies and rodents were recorded. A fraction of the caught sandflies was analyzed by PCR to detect *Leishmania* spp. Tissues from captured rodents were also screened for infection. Ecological Niche Models (ENMs) were computed for species of rodent and their association with crop-growing areas. We found 13 species of sandflies, several of which are first records for this region. Medically important species such as *Lutzomyia anthophora*, *Lutzomyia diabolica*, *Lutzomyia cruciata*, and *Lutzomyia shannoni* were documented. *Leishmania* spp. infection was not detected in sandflies. Nine species of rodents were recorded, and *Leishmania* (*Leishmania*) *mexicana* infection was found in four species of *Peromyscus* and *Sigmodon*. ENMs showed that potential distribution of rodent pest species overlaps with allocated crop areas. This shows that *Leishmania* (*L*.) *mexicana* infection is present in the Northeastern region of Mexico, and that previously unrecorded sandfly species occur in the same areas. These findings suggest a potential risk of transmission of *Leishmania* (*L.*) *mexicana*.

## Abstract (fr)

Les leishmanioses sont un groupe de maladies importantes transmises aux humains par piqûre des phlébotomes vecteurs. Plusieurs formes de leishmanioses sont endémiques au Mexique, surtout dans la région du sud-est. Dans la région du nord-est, cependant, il n’y a eu que des rapports isolés de cas et quelques signalements de phlébotomes. L’objectif principal de cette étude était d’analyser la diversité des phlébotomes et des hôtes-réservoirs potentiels de *Leishmania* spp. dans les états de Nuevo León et Tamaulipas. La richesse en espèces et l’abondance des phlébotomes et des rongeurs ont été étudiées. Une fraction des phlébotomes capturés a été analysée par PCR pour détecter les *Leishmania* spp. Les tissus des rongeurs capturés ont également été criblés pour détecter une infection. Les modèles de niches écologiques (ENM) ont été calculés pour les espèces de rongeurs et leur association avec les zones cultivées. Nous avons trouvé treize espèces de phlébotomes, dont plusieurs sont des premières mentions pour cette région. Des espèces médicalement importantes telles que *Lutzomyia anthophora*, *Lutzomyia diabolica*, *Lutzomyia cruciata* et *Lutzomyia shannoni* ont été documentées. L’infection par *Leishmania* spp. n’a pas été détectée chez les phlébotomes. Neuf espèces de rongeurs ont été collectées, et une infection par *Leishmania (Leishmania) mexicana* a été trouvée chez quatre espèces de *Peromyscus* et *Sigmodon*. L’ENM a montré que la répartition potentielle des espèces de rongeurs chevauche les zones allouées aux cultures. Cela montre que l’infection à *Leishmania (L.) mexicana* est présente dans la région nord-est du Mexique et que des espèces de phlébotomes précédemment non mentionnées se trouvent dans les mêmes zones. Ces résultats suggèrent un risque potentiel de transmission de *Leishmania (L.) mexicana*.

## Introduction

Human leishmaniases represent a health problem in many countries worldwide. It is estimated that the diseases are prevalent in at least 98 countries, with 1.5–2.0 million persons infected and over 350 million persons living at risk [1]. In Mexico, four clinical forms of the disease have been reported, localized cutaneous leishmaniasis (LCL), diffuse cutaneous leishmaniasis (DCL), mucocutaneous leishmaniasis (MCL), and visceral leishmaniasis (VL), with LCL being by far the most common clinical manifestation [88]. Transmission of *Leishmania* spp. to humans occurs by the infectious bites of several species of the genus *Lutzomyia* França [4, 57, 59, 73]. Up until now, most of the field studies concerning LCL in Mexico were conducted in the Yucatan Peninsula. These studies include entomological aspects [67–70], reservoir hosts [5, 83, 84], and clinical approaches [2, 30, 85].

Traditionally it has been assumed that the only proven vector of *Leishmania* (*L.*) *mexicana* in Mexico is the sandfly *Lutzomyia olmeca olmeca* (Vargas and Díaz-Nájera) [4], although in more recent studies, we have found evidence in the Yucatan Peninsula that other species such as *Lu. cruciata* (Coquillett), *Lutzomyia shannoni* (Dyar), and *Lutzomyia panamensis* (Shannon) may actually be acting as vectors as well [57, 59, 73]. These entomological studies were carried out in the Yucatan Peninsula. Unfortunately, there has been very little research in other regions of the country, even though cases of leishmaniasis have been reported there. A recent publication by González-Rosas et al. [26] revealed using Ecological Niche Modeling that the distribution of leishmaniasis in Mexico does not correlate well with the distribution area of the proven sandfly vector *Lu. olmeca olmeca*. These findings clearly suggest that other sandfly vectors – and possibly other reservoir hosts – are implicated in the transmission cycle in other foci of leishmaniasis.

To date, studies of leishmaniasis in Northeastern Mexico (NEM) have been very scanty and composed mainly of old records scattered across different geographical areas. From the clinical perspective, perhaps the first documented autochthonous case of leishmaniasis in NEM was reported by Ramos-Aguirre [64] in a six-year-old girl from the state of Coahuila. A case of DCL was identified in San Benito, Texas, USA [75]. However, in this study, it could not be established whether or not the case was autochthonous to the USA, as the 64-year-old infected woman reported frequent visits to the Mexican states of Nuevo León and Tamaulipas. In the state of Coahuila, Ramos-Aguirre [65] reported two additional cases of DCL: one was a 24-year-old male from the county of Múzquiz, whereas the second case was a 23-year-old male from the same county. At the University Hospital in Monterrey, state of Nuevo León, Welsh [89] reported one case of LCL in a 2-year-old male toddler who had always lived in the county of San Carlos, state of Tamaulipas. Several years later, Velasco-Castrejón et al. [88] reported six additional cases of DCL in Mexico; of those, three were from the NEM region: one from Tamaulipas (patient no. 4: a 15-year-old male), and two from the state of Coahuila (patient no. 5: a 40-year-old male and patient no. 6: a 17-year-old male). González-Piñeyro et al. [25] reported an 8-year-old patient from Nuevo León. Finally, a more recent report of cutaneous leishmaniasis concerns an unpublished case treated at the University Hospital in the city of Monterrey, corresponding to a 38-year-old male from the town of Méndez, Tamaulipas. So far, there have been at least seven cases in NEM reported officially; in addition; two recent suspected cases are being considered from the county of Guadalupe, in the state of Nuevo León (Dr. Nancy Treviño of the Health Secretariat of Mexico, per. com.).

Up to now, sandfly occurrences in NEM have been very limited and collection data are available in only a few publications [e.g. 16, 24, 86, 92]. The oldest known specimens are from collections made in 1936 in Tamaulipas by C. Plumer (see [86], for collection details). In addition to the published records, there are a small number of unpublished collections carried out by Rebollar-Téllez from 1993 and 2010. Adding up all these sandfly records, we determined that 52 specimens (34 ♂ and 18 ♀) of only five sandfly species (*Lu. anthophora* (Addis), *Lu. cruciata*, *Lu. diabolica* (Hall), *Lutzomyia oppidana* (Dampf), and *Lutzomyia texana* (Dampf)) have been recorded in nine different localities. It is therefore clear that there has been very little accumulation of knowledge on sandfly species in NEM over the last 74 years (1936–2010).

Similarly, knowledge on reservoir hosts in NEM is basically nil, and the only relevant paper is that of [94], in which the authors reported a 6-year-old English Bull Terrier infected with visceral leishmaniasis. This report is not likely to be an autochthonous case as the dog was brought to Mexico from Aragón in Spain.

The results we present here are in fact part of a larger network of multi-disciplinary and inter-institutional collaborations in Mexico concerned with zoonoses and, in particular, leishmaniasis. This network has several nodes in different geographical areas throughout the country. This particular study was conducted in the northern node of this network. Having observed that the available information on leishmaniases was so scattered (spatially and temporally), the lack of information about vectors and potential reservoir hosts and their distributions, coupled with the increasing number of clinical cases of leishmaniasis occurring in the southern USA, we decided to conduct this study. Due to the aforementioned facts, in the present work we hypothesized that the distribution of phlebotomine sandflies and rodents in Northeastern Mexico has not been properly documented, and in the context of an ecological niche, it is predicted that there is an association between vector species and reservoir hosts of *Leishmania* spp. Therefore, we began a systematic field study with the following objectives: (i) to analyze the diversity of phlebotomine sandfly and small rodents in Northeastern Mexico, (ii) to search for *Leishmania* spp. infection among samples of sandflies and rodents in Northeastern Mexico, and (iii) to develop a model for the potential distribution of trapped rodents using ENMs to estimate the potential risk of *Leishmania* spp. disease when these hosts become crop pests.

## Material and methods

### Description of study sites

**Figure 1.**
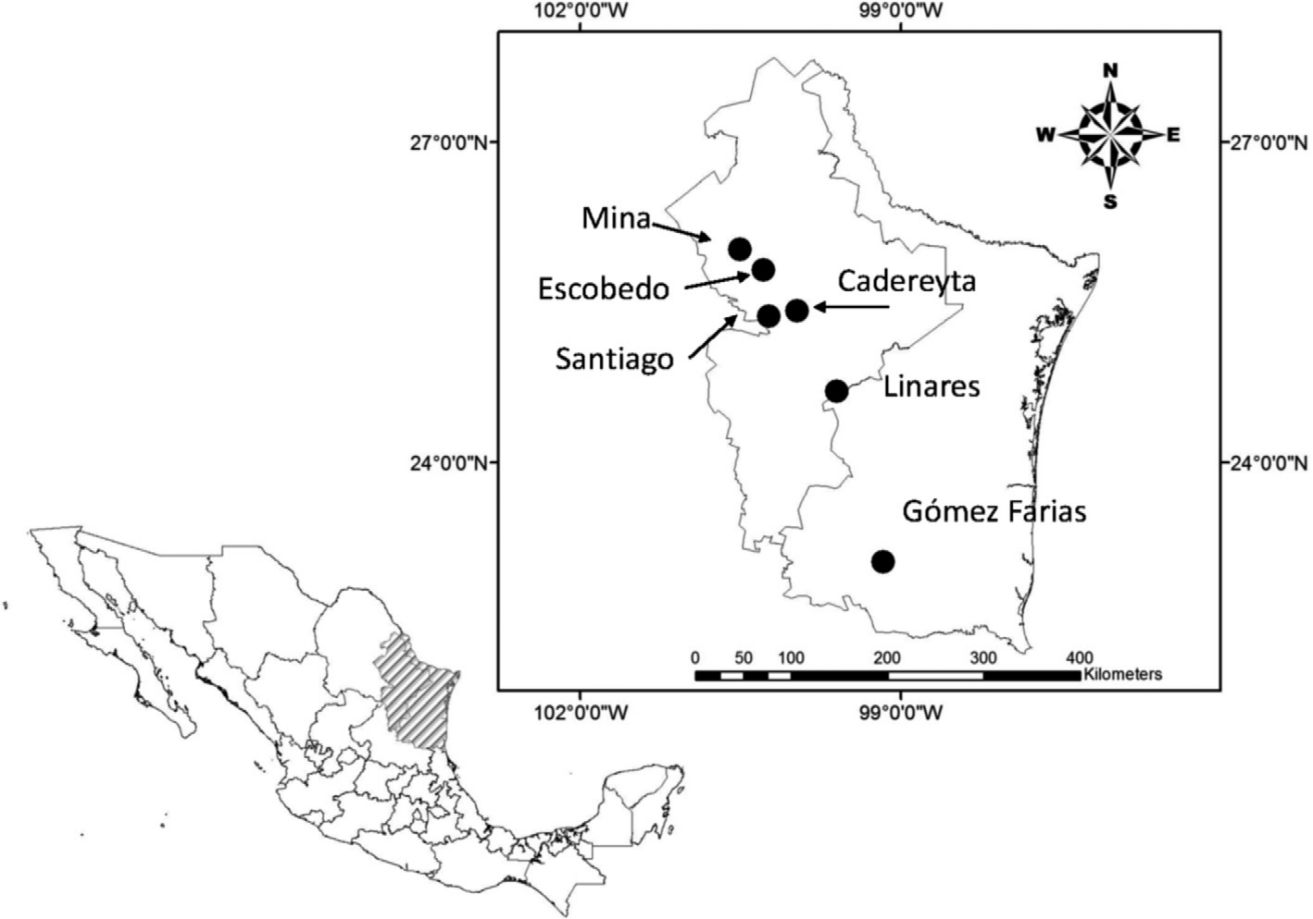
Geographical location of the sampling sites in the states of Nuevo León and Tamaulipas, Mexico.

**Figure 2.**
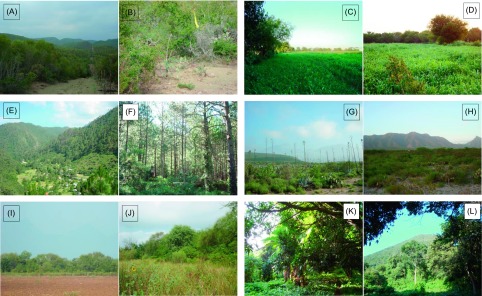
General landscape views of study sites in the states of Nuevo León and Tamaulipas. Nuevo León: Linares, Rancho San Manuel (A and B); Escobedo, Ejido San Nicolás, Predio Colectivo Viejo (C and D); Santiago, Ciénega de González (E and F); Mina, Ejido Labores del Ojo (G and H); Cadereyta Jiménez, Fraccionamiento Rincón de los Sabinos Second Sector (I and J); and the state of Tamaulipas: Gómez Farías (K and L).

During the period between April and August 2010, we conducted fieldwork in five different localities in the states of Nuevo León (Linares, Escobedo, Santiago, and Cadereyta Jiménez) and Tamaulipas (Gómez Farías) (Table 1, Fig. 1). Biogeographically, the states of Nuevo León and Tamaulipas are considered to be part of the Nearctic region with four different provinces: Altiplano, Tamaulipas, Sierra Madre Oriental, and Gulf of Mexico [55]. A brief description of each study site is as follows. Linares: weather is classified as semi-warm dry (BS_1_hw) with summer rains, mean annual temperature 22 °C, and a total rainfall of 749 mm [23]. Vegetation in the area is composed of sub-mountain shrubs, mainly *Helietta parvifolia* Gray, *Prosopis glandulosa* Torr, *Acacia farnesiana* Linnaeus, and *Acacia rigidula* Benth [71] (Figs. 2A and 2B). Escobedo: weather is warm dry (BS_1_(h′)h), mean annual temperature of 22–24 °C, and a total rainfall of 400–600 mm [23]. This site is an agricultural area of sorghum and oak with remnant trees *Prosopis glandulosa*, *Ehretia anacua* Teran and Berland, *Parkinsonia aculeata* Linnaeus, and *Pithecellobium ebano* Berland [71] (Figs. 2C and 2D). Santiago: weather is classified as semi-warm humid ((A) C_1_a), the mean annual temperature is 20.9 °C, with an annual rainfall of 1015 mm [23]. This site is located in the Ciénega de González forest of the mountain chain known as the Sierra Madre Oriental. Dominant tree species are pines and oaks, such as *Quercus rysophylla* Weath, *Quercus laeta* Liebm, *Quercus polymorpha* Schlecht and Cham, *Pinus teocote* Schlecht and Cham, and *Pinus pseudostrobus* Lindl [71] (Figs. 2E and 2F). Mina: weather is classified as warm dry (BS_1_(h′)h) with an annual temperature of 24 °C and an annual rainfall of 270 mm [23]. Vegetation at this site is composed of xerophytes: *Acacia berlandieri* Benth, *Cordia boissieri* A. DC., *Agave lechuguilla* Torrey, *Prosopis glandulosa*, and several species of *Opuntia* spp. [71] (Figs. 2G and 2H). Cadereyta Jiménez: weather is semi-warm dry (BS_1_hw), with an annual temperature of 23 °C and an annual rainfall of 601–800 mm [71]. The study site is composed of suburban settlements surrounded by agriculture and secondary patches of vegetation containing *Prosopis glandulosa*, *Acacia farnesiana*, *Ehretia anacua*, and *Pithecellobium ebano* [71] (Figs. 2I and 2J). Gómez Farías: since this site is located in the transition between the Nearctic and Neotropical biotic provinces, it is classified as semi-warm humid ((A)C_1_), the mean annual temperature is 21 °C with an annual rainfall of 1558–1778 mm [23]. Tree species are *Mangifera indica* Linnaeus, *Ceiba pentandra* Linnaeus, *Brosimum alicastrum* Swartz, and *Mirandaceltis monoica* Greene [71] (Figs. 2K and 2L).

**Table 1.** Name and geographic location of the selected sampling sites in the states of Nuevo León and Tamaulipas, Mexico. Collection dates at each site are also listed.

State	Municipality	Location	Date	Altitude (m asl)	Coordinates
Nuevo León	Linares	Rancho San Manuel	06–08/Apr/2010	446	24°40′08″ N; 099°36′16″ W
	Escobedo	Ejido San Nicolas, Predio Colectivo Viejo	05–07/May/2010	447	25°48′22″ N; 100°17′17″ W
	Santiago	Cienega de Gonzalez	20–22/Jun/2010	1304	25°22′39″ N; 100°14′31″ W
	Mina	Ejido Labores del Ojo	12–14/Jul/2010	574	26°00′04″ N; 100°30′40″ W
	Cadereyta Jiménez	Fraccionamiento Rincon de los Sabinos 2° Sector	27–29/Aug/2010	325	25°25′35″ N; 099°58′23″ W
Tamaulipas	Gómez Farías	Gómez Farías	28–30/Jul/2010	347	23°04′17″ N; 099°10′16″ W

*Note*. m asl: meters above sea level.

### Sandfly collection and processing

Phlebotomine sandflies were captured using CDC light traps^®^ (model 512; John W. Hock Co., Gainesville, FL, USA) [81]. Each CDC light trap was hung at a height of 1.5 m above the ground. These traps were operated from 18:00 to 07:00 h. A total 16 CDC light traps were used per night at each site, where, depending on the features of the site, we distributed the traps along a single straight transect or following the contours of a creek. In all cases, traps were positioned at 25 m intervals from each other. We also used six aluminum trays (50 × 50 cm), known as Disney traps [17], per night at each site, which were coated with a thin layer of castor oil and baited with BALB/c female mice. Each morning, captured sandflies were placed in an airtight container and a small ball of cotton wool was impregnated with ether as a killing agent. Specimens were then sorted and separated from other insects collected in the same traps. All caught insects were placed onto a white plastic tray. A pair of fine tweezers was used to separate sandfly specimens that were then preserved in plastic vials containing 70% ethanol. Approximately 20% of the caught females were individually preserved in 250 μL Eppendorf vials containing 200 μL of grade analysis ethanol. On arrival at the laboratory facilities, all samples were kept in a −20 °C freezer until they were processed. Sandfly processing was conducted following standard curatorial techniques [34, 92], while identification was carried out using as a reference Young and Duncan [93] and Ibáñez-Bernal [35, 36]. Phlebotomine sandflies species reported herein follow the classical nomenclature system of Lewis et al. [46]. Voucher specimens are held at the Medical Entomology Laboratory (UANL).

### Rodent collection and processing

Rodents were only collected at localities in the state of Nuevo León. No collections were made in Gómez Farías, Tamaulipas due to heavy rains. Small rodents were collected using 30 collapsible live Sherman traps^®^ per night. Each trap was baited with a mix of oat flakes impregnated with vanilla essence. All traps were set at ground level at intervals of 25 m on a single transect per site. All captured rodents were taxonomically identified and sexed. For these individuals, we also recorded standard body measurements and weight. Each individual was sacrificed and processed to obtain tissue samples of the skin surrounding the tail, biopsies of the ears, liver, spleen, and heart. All tissues were coded and were preserved in 2 mL sterile Eppendorf vials containing 70% ethanol. All tissues were kept in a −20 °C freezer until they were processed for molecular analyses. For the analysis of *Leishmania* infection among rodent species, we included specimens captured at several collections conducted in the state of Nuevo León; however, to compare diversity among sites, we only included those sites with the same trapping effort. Specimens were handled and euthanized according to the guidelines of the American Society of Mammalogists for the use of wild mammals in research [22], and under a collecting permit issued by the General Directorate of Wildlife of Mexico (permission number SGPA/DGVS/00471/11). Voucher specimens are held at the Medical Entomology Laboratory (UANL).

### Analysis of diversity in sandfly and rodent assemblages

For alpha diversity, Chao1-bc (bias-corrected form for the Chao1 estimator) [6, 7], exponential of Shannon entropy index, and inverse of Simpson concentration index were estimated and called Hill numbers [31] with order *q* = 0, 1, 2, respectively. The diversity of order *q* indicates its sensitivity to common and rare species [38]. The diversity of order zero (*q* = 0) is completely insensitive to species abundance and is known as species richness of an assemblage; when *q* = 1, the weight for all species is mainly due to their abundance and therefore does not favor either common or rare species; and when *q* = 2, the major weight is placed on the most common species [38]. These indices have the intuitive properties (principle of duplication and replication) expected of diversity [38–40]. Hill numbers unify the diversity measured combining several in one expression called “effective number of species” that allows us to compare the magnitude of the difference in the diversity of two or more communities [31, 38, 47]. The estimator Chao1-bc (*q* = 0) uses only the numbers of singletons and doubletons to estimate the number of undetected species in the sample [8]. Individual-based abundance data and the Maximum Likelihood Estimator (MLE) were used. A bootstrap procedure of 100 replications was applied to obtain variances and to construct the confidence intervals of estimated diversities. These three estimates were calculated by the online program SpadeR (Species Prediction and Diversity Estimation) [7]. For Clench’s equation [12], it estimates the total number of species in relation to trapping effort. The software program was set at 100 randomizations of the dataset before analysis in EstimateS version 9.1.0 [13]. Then, nonlinear regressions with Simplex and Quasi-Newton logarithms in STATISTICA software, v. 10.0 (StatSoft Inc. USA) were used in this equation [37, 76]. For beta diversity, Jaccard’s similarity index was used, and the binary data (presence/absence) were analyzed using the Multi-Variate Statistical Package (MVSP v. 3.22). Using Jaccard’s coefficients, a dendrogram was constructed by Unweighted Pair Group Method Average (UPGMA).

The co-occurrence of rodent and sandfly species was evaluated using a C-Score model [80] with the software EcoSim (v. 7.72) [28]. The C-Score model requires data on presence (1) and absence (0), and before analyses 5,000 randomizations were carried out. If the observed value of the model is greater than the simulated value, then a segregated pattern is established. In contrast, if the observed value is less than the simulated model, then an aggregated pattern is established. Finally, if the observed and simulated values are equal, then a random co-occurrence is established [9, 10]. A *Z*-statistic was calculated to test the null hypothesis that sandfly sex proportions were equal in the samples. To test whether there was a significant association between species (insects and rodents) and the study sites, we compared the relative abundances by χ^2^ analyses using a *c* × *r* table. All statistical tests were considered significant if *p* < 0.05 [76].

### Molecular analysis of *Leishmania* strains in sandfly and rodent samples

DNA from rodent tissues was extracted from approximately 25 mg of tissues, using a commercial DNA extraction kit (DNeasy^®^ Blood and Tissue kit, Qiagen, Hilden, Germany), following the manufacturer’s instructions. The DNA was analyzed by PCR. For sandfly analysis, the abdomen and thorax of individual females were used for DNA extraction, using a modified plasmid extraction Sambrook protocol [72] and Pech-May et al. method [58]. The DNA pellets were resuspended in 30 μL distilled water and 50 ng DNA was subjected to PCR amplification.

To determine the presence of *Leishmania*, we used oligonucleotides based on the *Leishmania* mini-circle kinetoplast DNA, L.MC-1S, and L.MC-1R [41]. Identification of *Leishmania* (*L.*) *mexicana* species was done using the small subunit of the 18S ribosomal gene as forward primer IR1 (designed by Cupolillo [15]), and the internal transcribed spacer of the ribosomal RNA (rRNA) gene as reverse primer LM17 [3]. Amplification reactions were done in 50 μL of reaction mixture: Taq PCR Master Mix (Qiagen, Hilden, Germany), 100 ng of the corresponding oligonucleotides, and 1 μL of tissue extract corresponding to 100 ng of DNA, or 50 ng of DNA from *Lutzomyia*. The amplification was carried out in a Perkin Elmer 2720 thermocycler using different conditions, depending on the oligonucleotides used. For L.MC-1S/L.MC-1R (*Leishmania* genus), 30 cycles at 95 °C for 1 min (denaturation), 55 °C for 1 min (annealing), and 72 °C for 1 min (polymerization) were used. For IR1/LM17 (*Leishmania* (*L.*) *mexicana*), 35 cycles at 94 °C for 1 min, at 65 °C for 1 min, and at 72 °C for 1 min were used. In all cases, the cycles were preceded by a cycle at 94 °C for 5 min and a final extension cycle at 72 °C for 7 min. As a positive control for *Lutzomyia* DNA extraction, a conserved region of the 18S rRNA gene (450 bp) was used. PCR was carried out using Lu.18S rRNA-1S (5′-TGCCAGTAGTTATATGCTTG-3′) and Lu.18S rRNA-1R (5′-TTACGCGCCTGCTGCCTTCC-3′) [41]. The amplification conditions were the same as described for *Leishmania* genus. PCR products were analyzed in 1.5% agarose gel electrophoresis in Tris-acetate-EDTA (TAE) buffer at 80 V, stained with 0.5 μg/mL ethidium bromide, and visualized under ultraviolet light.

### Analysis of Ecological Niche Modeling of rodent and sandfly distribution

We built the ecological niche of infected rodents and sandflies and projected them to generate the potential distribution models of the infected-rodent and sandfly species (IRSS) captured. We modeled IRSS potential distribution to determine the geographic and ecological extent of leishmaniasis using as predictors both rodents confirmed as infected by *Leishmania* (by this study) and potential vectors. With this approach, we can provide information about a large unsampled region. We used the Global Biodiversity Information Facility (GBIF) public database and the occurrence data of the different species provided by the laboratory of Geographic Information Systems of the National Autonomous University of Mexico. We modeled only those IRSS that have more than 10 independent georeferenced data, and used nine WorldClim bioclimatic variables that could determine the IRSS’ presence (www.worldclime.com). Variables used were: annual mean temperature, temperature seasonality, mean temperature of warmest quarter, mean temperature of coldest quarter, annual precipitation, precipitation of the driest month, precipitation seasonality, precipitation of driest quarter, and altitude. As regional models tend to overfit the data, we used only the biogeographic provinces that covered our region of interest (i.e. NEM and the Southeast of the USA). To implement the models, we used the MaxEnt algorithm, which has proven to be robust in the predictions of the potential distribution of species [54]. To build the models, 75% of data were used to train the models and 25% were used as test data. We created 100 replicates, then all models generated were analyzed one by one, and the best model for each species was chosen. The accuracy of each model was assessed using both the AUC (area under the receiver operating characteristic [ROC] curve), which is automatically generated by MaxEnt, and the 11 binomial tests of model performance, which are reported as part of the MaxEnt output. All 11 binomial tests were required to be significant at a confidence level of *p* < 0.01, which is also a conservative choice. We considered only those niche models possessing both a *p* value less than 0.01 for the binomial test of omission and an AUC greater than 0.80. It is a well-known fact that several rodent species have the potential to become crop pests, thereby increasing the risk of transmission to humans. To test whether or not there was an association between crop areas and the distribution of rodents, we overlapped the map of the three main crops in the region: sorghum, bean, and barley on the ENM of all rodents registered at the study sites.

## Results

### Sandfly diversity

During the sampling period (April to August 2010), we collected a total 724 specimens of sandflies comprising 13 species in two genera (*Lutzomyia* and *Brumptomyia* França and Parrot). Out of the total 724 sandflies, 11.88% (46 ♀ and 40 ♂) were collected in the state of Nuevo León and 88.12% (325 ♀ and 313 ♂) in the state of Tamaulipas (Table 2). We were able to collect sandfly specimens at all the selected sites with the exception of Mina. The most abundant species in the state of Nuevo León were *Lu. texana* (37.21%) and *Lu. diabolica* (27.91%). In the locality of Cadereyta Jiménez, we observed the highest species richness 6.96 (6.07**─**19.36), and 4.22 (3.63**─**4.81) effective species in the state of Nuevo León (Table 2). We also observed that in the same locality, the species *Brumptomyia hamata* (Fairchild and Hertig) represented a singleton (1 ♂) (Table 2). Approximately 95% of specimens were collected with CDC light traps, and the rest with Disney traps. Using a *Z*-statistic to test the hypothesis of equal proportion of sex, we concluded that the sex proportion (1:1 female:male) was not significantly different for the captures in Nuevo León (females 53.49% versus males 46.51%) (*Z* = −1.16, *p* < 0.27). At Gómez Farías, in the state of Tamaulipas, we found that *Lu*. *shannoni* (66.30%) and *Lu*. *cruciata* (30.41%) were the most common species. On the other hand, a singleton was observed for *Lutzomyia trinidadensis* (Newstead) (1 ♂). Although the majority of specimens were collected in CDC light traps, we also captured female sandflies (28.37%) that were attracted to humans in Tamaulipas. We also tested the null hypothesis of equal proportions (female:male) in the catches carried out in the state of Tamaulipas (females 50.94% versus males 49.06%), finding that there was no significant difference (*Z* = −1.01, *p* = 0.31).

**Table 2.** Species composition and total abundance and relative abundance (%) of male and female phlebotomine sandflies caught in Nuevo León and Tamaulipas, Mexico. Estimated diversities of Chao1-bc (*q* = 0) (bias-corrected form for the Chao1 estimator), exponential of Shannon entropy index (*q* = 1), and inverse of Simpson concentration index (*q* = 2), with its confidence intervals based on a bootstrap method of 100 replications. Collections were conducted from April to August 2010.

	Nuevo León								Tamaulipas		
	Linares		Escobedo		Santiago		Cadereyta		Gómez Farías		
Species	♂	♀	♂	♀	♂	♀	♂	♀	♂	♀	Total (%)
*Brumptomyia hamata*	0	0	0	0	0	0	1	0	0	0	1 (0.14)
*Brumptomyia mesai*	0	0	0	0	0	0	2	3	0	0	5 (0.69)
*Lutzomyia anthophora*	0	0	0	3	0	0	0	6	1	0	10 (1.38)
*Lutzomyia cratifer*	0	0	0	0	0	0	0	0	2	6	8 (1.10)
*Lutzomyia cruciata*	0	1	0	0	0	0	0	1	1	193	196 (27.07)
*Lutzomyia ctenidophora*	0	0	0	0	0	0	0	0	0	2	2 (0.28)
*Lutzomyia diabolica*	0	0	0	0	14	10	0	0	0	0	24 (3.31)
*Lutzomyia leohidalgoi*	0	0	0	0	0	0	0	0	0	2	2 (0.28)
*Lutzomyia oppidana*	0	0	0	0	0	0	0	0	2	3	5 (0.69)
*Lutzomyia shannoni*	0	0	0	0	1	0	0	0	306	117	424 (58.56)
*Lutzomyia texana*	8	7	0	0	0	0	6	11	0	2	34 (4.70)
*Lutzomyia trinidadensis*	0	0	0	0	0	0	0	0	1	0	1 (0.14)
*Lutzomyia vindicator*	0	2	0	0	0	0	8	2	0	0	12 (1.66)
Number of individuals	8	10	0	3	15	10	17	23	313	325	724 (100)
Number of species	3		1		2		6		9		13
Estimator Chao1-bc	3.00 (3.00**─**5.00)		1.00 (1.00**─**1.00)		2.00 (2.00**─**2.00)		6.96 (6.07**─**19.36)		9.25 (9.00**─**13.80)		13.33 (13.00**─**19.00)
Exponential of Shannon entropy index	1.75 (0.97**─**2.52)		1.00 (1.00**─**1.00)		1.18 (0.89**─**1.47)		4.22 (3.63**─**4.81)		2.23 (2.09**─**2.37)		3.39 (3.06**─**3.72)
Inverse of Simpson concentration index	1.41 (0.80**─**2.02)		1.00 (1.00**─**1.00)		1.08 (−1.58**─**3.75)		3.54 (2.77**─**4.31)		1.88 (1.78**─**1.98)		2.38 (2.19**─**2.56)

**Figure 3.**
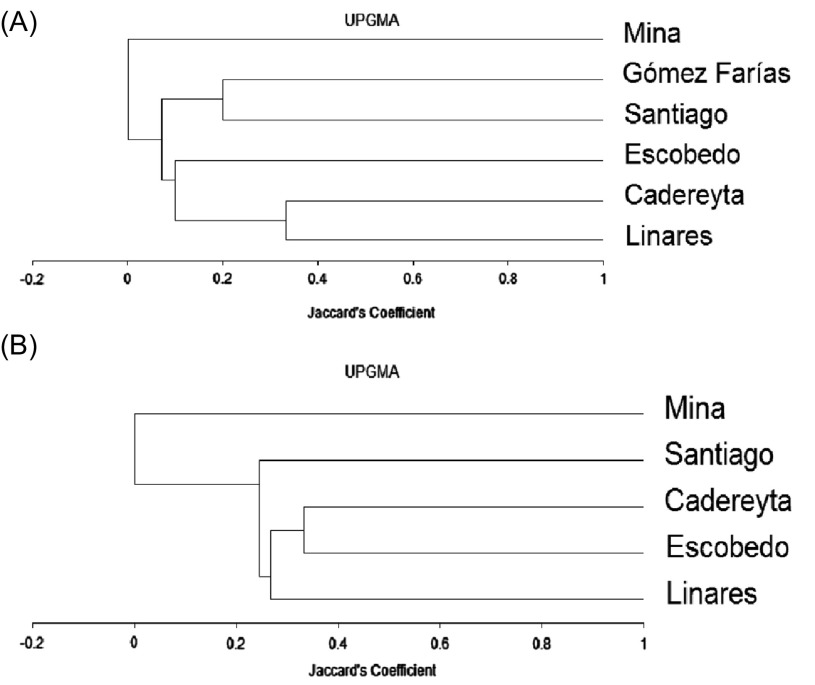
(A) Dendrogram according to Jaccard’s similarity coefficient from phlebotomine sandflies, and (B) rodents collected in the states of Nuevo León and Tamaulipas, Mexico.

**Figure 4.**
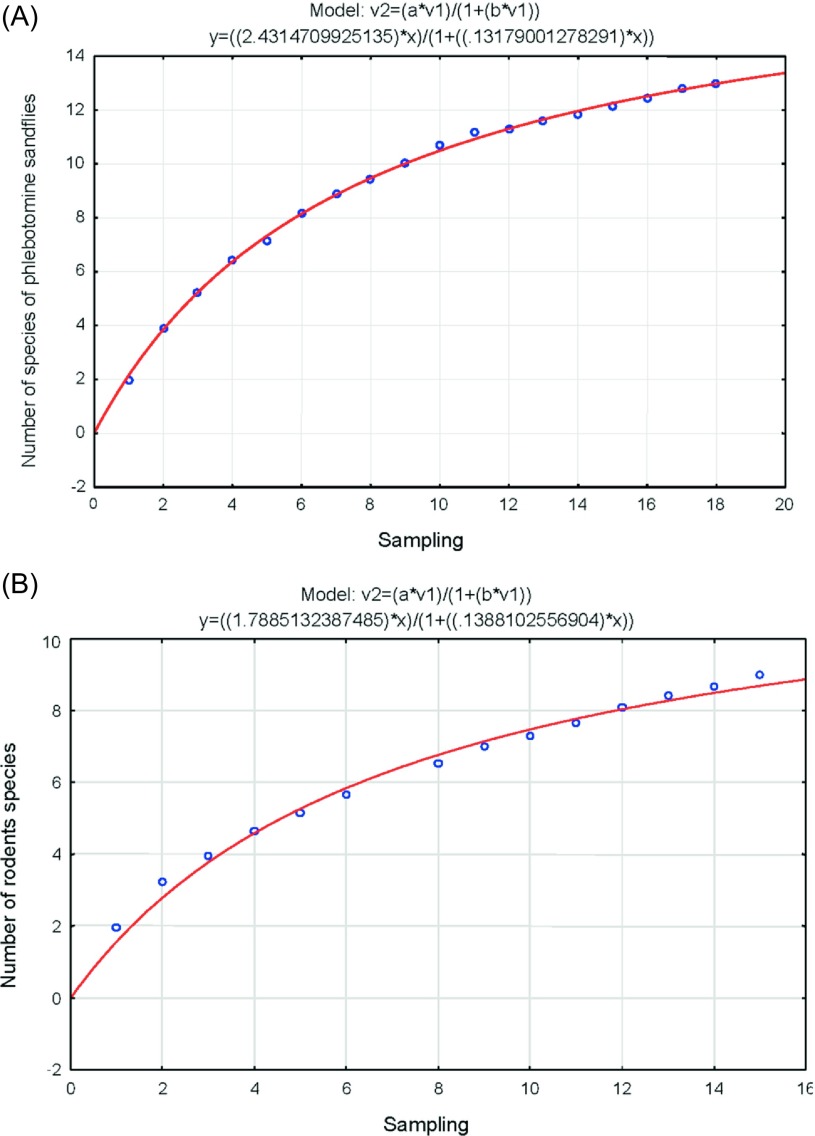
(A) Accumulation curve of species of phlebotomine sandflies, and (B) rodents species in Nuevo León and Tamaulipas, Mexico.

Beta diversity using Jaccard’s index indicated that the study sites of Linares and Cadereyta Jiménez were the most similar in terms of phlebotomine sandfly species (33% and 50%), respectively. The less similar study sites were Cadereyta Jiménez and Gómez Farías (Fig. 3A). Using combined data from Nuevo León and Tamaulipas for sandfly species abundances, we calculated the key parameters in Clench’s equation. We found that the observed data fit well the expected values of the curves of species accumulation in relation with trapping effort. Nonetheless, we found that accumulation of species did not reach the predicted asymptote. Based on the intercept value (*a* = 2.43) and the slope value (*b* = 0.13), it was estimated that sandfly trapping effort was 70% and that we recorded 13 species out of the 18 predicted species (Fig. 4A). We also observed that sandfly species abundances were statistically associated with study sites, (χ^2^ = 177.88, *df* = 52, *p* < 0.05), indicating a heterogeneity in spatial distribution in sandfly species richness. We found for sandfly species that the *C*-score value was 0.71, as compared with 0.78 (± 0.003) of the mean (± variance of simulated *C*-score values) (*p* = 0.16). Estimations of the *C*-score show that the species exhibited neither an aggregated nor a segregated pattern.

### Rodent diversity

A total 79 specimens of nine species were collected in all sampled sites. The most abundant species were *Peromyscus maniculatus* (Wagner) (37.97%), *Sigmodon hispidus* Say and Ord (30.38%), and *Peromyscus leucopus* Rafinesque (16.46%). The highest species richness and abundances were found in Cadereyta Jiménez (60.76%), where the most common species at this site were *S*. *hispidus* (41.66%), *P*. *maniculatus* (29.17%), and *P*. *leucopus* (16.66%). Sampled among the species “singletons” were *Dipodomys merriami* Mearns, *Heteromys irroratus* (Gray), [63], and *Peromyscus eremicus* (Baird) (Table 3). The total number of species in the state of Nuevo León represents 75% of the total species richness estimated with Chao1-bc. The locality of Cadereyta had 1.58 times more diversity than Linares, and a little more than twice that in Escobedo, Santiago and Mina (Table 3). In the case of beta diversity, we found using Jaccard’s index that Linares shared 25% similarity with Escobedo and Santiago; likewise, Escobedo shared 33% similarity with Santiago and Cadereyta Jiménez (Fig. 3B).

**Table 3.** Rodent species and abundances at each study site. Relative abundances are expressed as a percentage of the total rodent species caught. Numbers in this table include only those specimens collected at sites with the same trapping effort. Estimated diversities of Chao1-bc (*q* = 0) (bias-corrected form for the Chao1 estimator), exponential of Shannon entropy index (*q* = 1), and inverse of Simpson concentration index (*q* = 2), with its confidence intervals based on a bootstrap method of 100 replications. Sampling was conducted only at study sites in the state of Nuevo León. Collections were conducted from April to August 2010.

	Nuevo León										
	Linares		Escobedo		Santiago		Mina		Cadereyta		
Species	♂	♀	♂	♀	♂	♀	♂	♀	♂	♀	Total (%)
*Chaetodipus hispidus*	1	0	0	0	0	0	0	0	0	2	3 (3.80)
*Chaetodipus nelsoni*	0	0	0	0	0	0	2	1	0	0	3 (3.80)
*Dipodomys merriami*	0	0	0	0	0	0	0	1	0	0	1 (1.30)
*Neotoma micropus*	0	0	0	0	0	0	0	0	2	1	3 (3.80)
*Heteromys irroratus*	0	0	0	0	0	0	0	0	0	1	1 (1.30)
*Peromyscus eremicus*	1	0	0	0	0	0	0	0	0	0	1 (1.30)
*Peromyscus maniculatus*	3	0	7	0	3	3	0	0	7	7	30 (38.50)
*Peromyscus leucopus*	0	0	0	0	3	2	0	0	5	3	13 (16.70)
*Sigmodon hispidus*	0	0	3	1	0	0	0	0	10	10	24 (30.80)
Number of individuals	5	0	10	1	6	5	2	2	24	24	79 (100)
Number of species	3		2		2		2		6		9
Estimator Chao1-bc	4.00 (3.10**─**15.90)		2.00 (2.00**─**2.30)		2.00 (2.00**─**2.20)		2.00 (2.00**─**2.00)		6.00 (6.00**─**7.90)		12.00 (9.40**─**34.00)
Exponential of Shannon entropy index	2.59 (1.37**─**3.81)		1.93 (1.63**─**2.22)		1.99 (1.74**─**2.24)		1.76 (0.94**─**2.57)		4.09 (3.08**─**5.11)		4.78 (3.83**─**5.74)
Inverse of Simpson concentration index	2.27 (1.43**─**3.12)		1.86 (1.42**─**2.30)		1.98 (1.70**─**2.27)		1.60 (0.73**─**2.47)		3.42 (2.54**─**4.29)		3.73 (3.11**─**4.35)

Using the accumulation curve model of Clench’s, we found that data from the state of Nuevo León showed a good fit between observed and expected values. The key parameters of Clench’s equation were the intercept (*a* = 1.78) and the slope (*b* = 0.13). With these values, it was estimated that sampling effort was 69% with an observed number of species of nine and an expected number of 13 (Fig. 4B). It was found that abundances of rodents were spatially associated with study sites (χ^2^ = 204.52, *df* = 32, *p* < 0.05). We found for rodent species that value was 0.83, as compared with 0.95 (± 0.007) of the mean (± variance of simulated *C*-score values) (*p* = 0.07). Estimations of the *C*-score show that species exhibit neither an aggregated nor a segregated pattern.

### Phlebotomine sandflies and rodents analyzed by PCR

A total of 163 female sandfly specimens were individually analyzed by PCR, and were negative for *Leishmania* spp. Sandfly species tested were: *Brumptomyia mesai* (Sherlock) (*n* = 1), *Lu*. *anthophora* (*n* = 2), *Lutzomyia cratifer* (Fairchild and Hertig) (*n* = 1), *Lu*. *cruciata* (*n* = 98), *Lu*. *diabolica* (*n* = 1), *Lu. oppidana* (*n* = 2), *Lu*. *shannoni* (*n* = 48), *Lu*. *texana* (*n* = 8), and *Lutzomyia vindicator* (Dampf) (*n* = 2).

Tissues of collected rodents were tested by PCR to detect evidence of infection, finding that the species *P*. *eremicus* (*n* = 1, 100%), *P*. *leucopus* (*n* = 23, 21.05%), *P*. *maniculatus* (*n* = 36, 5.88%), and *S*. *hispidus* (*n* = 27, 12.50%) were positive for genus-*Leishmania* parasites. The use of species-specific primers IR1/LM17 revealed that the infecting parasites were *Leishmania* (*L*.) *mexicana* (Table 4). For additional confirmation that the infecting parasites were *Leishmania* (*L*.) *mexicana*, the PCR amplifications obtained with the primers IR1/LM17 were sequenced at the Molecular Biology Unit of the Institute of Cellular Physiology, UNAM. The sequences were aligned with those from the National Center for Biotechnology Information, US National Library of Medicine, Basic Local Alignment Search Tool (BLAST). The gene sequences of our study showed a 99% identity with genes reported in GenBank under the accession numbers: *Leishmania mexicana* strain MHOM/MX/94/INDRE NBO (AF466381.1); *Leishmania mexicana* strain MHOM/MX/85/SOLIS (AJ000313.1); *Leishmania mexicana* isolate 169 clone 1 (FJ948434.1); *Leishmania mexicana* strain MHOM/MX/84/SET GS (AF466380.1); *Leishmania mexicana* strain MHOM/MX/98/UNAM RR (AF466382.1); *Leishmania mexicana* strain MHOM/GT/86/GO22 (AJ000312.1); *Leishmania mexicana* isolate 7 clone 1 (FJ948433.1).

**Table 4.** Rodent species caught at several sites in the state of Nuevo León. Total numbers refer to the sample size analyzed by PCR for detection of *Leishmania* (*L.*) *mexicana*, whereas numbers between parentheses refer to the positive samples. Some specimens in this table were also obtained via sporadic collection.

Rodent species	Total	Prevalence (%)	Biopsies (+)	Sites of individuals
*Chaetodipus hispidus*	4 (0)	0	–	LI (*n* = 2), CJ (*n* = 2)
*Chaetodipus nelsoni*	3 (0)	0	–	MI (*n* = 3)
*Dipodomys merriami*	1 (0)	0	–	MI (*n* = 1)
*Neotoma micropus*	16 (0)	0	–	CJ (*n* = 3), BU (*n* = 13)
*Heteromys irroratus*	1(0)	0	–	CJ (*n* = 1)
*Perognathus flavus*	1 (0)	0	–	BU (*n* = 1)
*Peromyscus eremicus*	1 (1)	100.00	L	LI (*n* = 1)
*Peromyscus leucopus*	19 (4)	21.05	L, S, H	BU (*n* = 3), SA (*n* = 5), CJ (*n* = 8), LI (*n* = 3)
*Peromyscus maniculatus*	34 (2)	5.88	L, S, H	BU (*n* = 1), SA (*n* = 6), ES (*n* = 7), CJ (*n* = 14), LI (*n* = 6)
*Peromyscus pectoralis*	2 (0)	0	–	LI (*n* = 2)
*Reithrodontomys fulvescens*	1 (0)	0	–	BU (*n* = 1)
*Sigmodon hispidus*	24 (3)	12.50	L, H	CJ (*n* = 1), ES (*n* = 4), CJ (*n* = 20)
Total	107 (10)	9.35		

Abbreviation of biopsies: L: liver, S: spleen, and H: heart. Abbreviation of sites: LI: Linares, SA: Santiago, CJ: Cadereyta Jiménez, MI: Mina, ES: Escobedo, and BU: Bustamante.

### Ecological Niche Modeling of rodent and sandfly distribution

**Figure 5.**
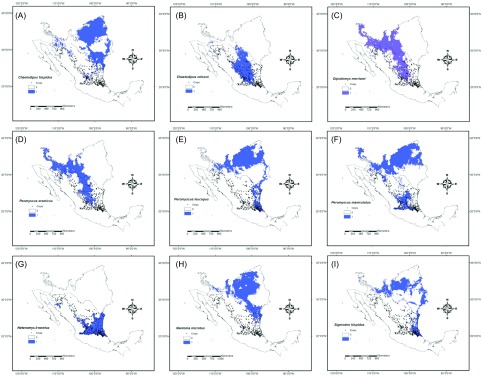
Ecological niche models of nine rodent species trapped and the geographic relation with crops in the Mexican area.

**Figure 6.**
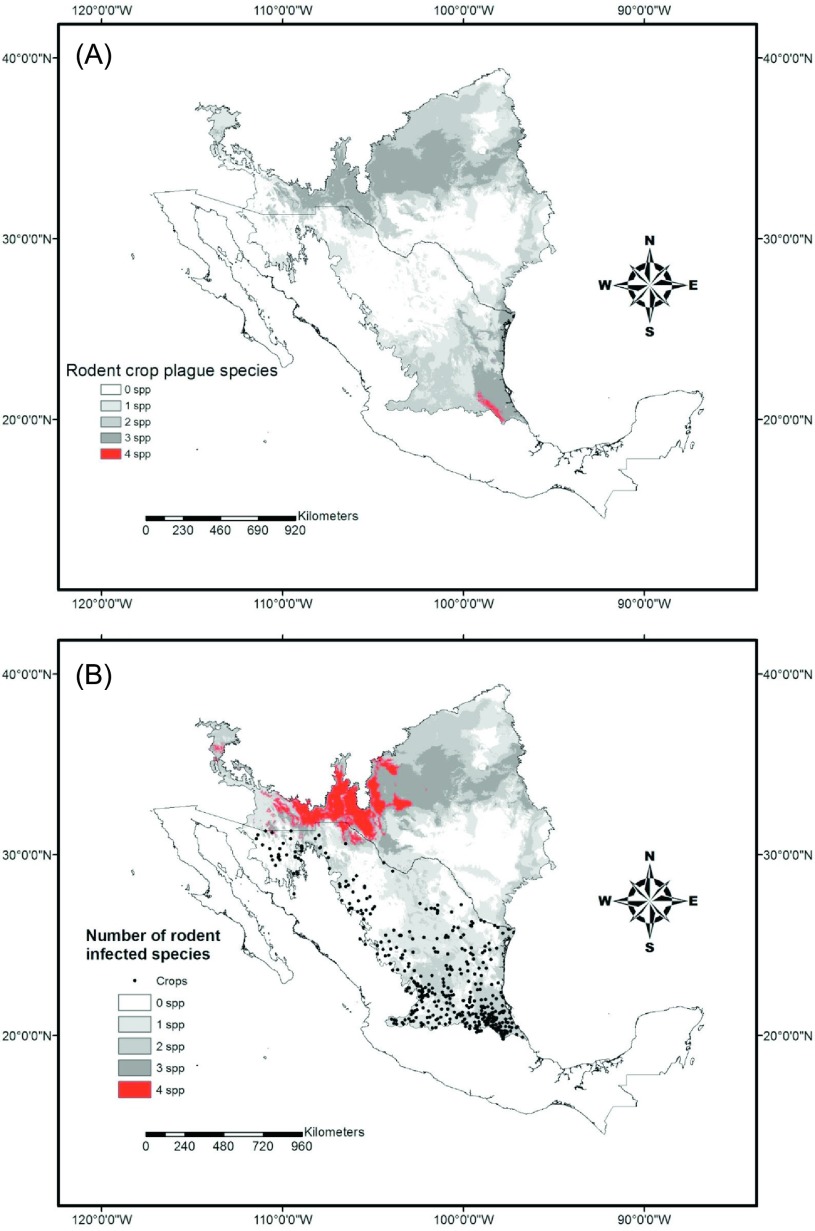
(A) Ecological niche models showing the geographic coincidence of the four rodent species caught during the study considered as crop plagues, and (B) the geographic coincidence of *Leishmania-*infected rodent species.

All nine rodent ENMs constructed had an average AUC of 0.90 ± 0.0002 and an accuracy that ranged from 0.80 to 0.97. The observed distribution of nine rodents showed variation in their potential distribution. *Chaetodipus* species have a complementary potential distribution. *Chaetodipus hispidus* (Baird) possesses a northern distribution while *Chaetodipus nelsoni* Merriam has a southern distribution (Fig. 5). *Peromyscus* spp. also showed a potential distribution that covers the entire study area (Fig. 5). *H*. *irroratus* showed a southern distribution, while *S. hispidus* has a wide distribution nationwide. In particular for the study area, its distribution is related to the presence of crops. The species *D*. *merriami* and *N*. *micropus* showed a more northwestern distribution (Fig. 5). The four species considered crop pests showed a generalized regional potential distribution. According to the potential distribution models, the four species have similar occurrences in the southern part of the study area (Fig. 6A). Three of these four species (*P. leucopus*, *P. maniculatus*, and *S. hispidus*) were positive to *Leishmania* spp. parasites; these plus *P*. *eremicus*, also positive, showed a geographical coincidence in the northwestern part of the study area (Fig. 6B).

**Figure 7.**
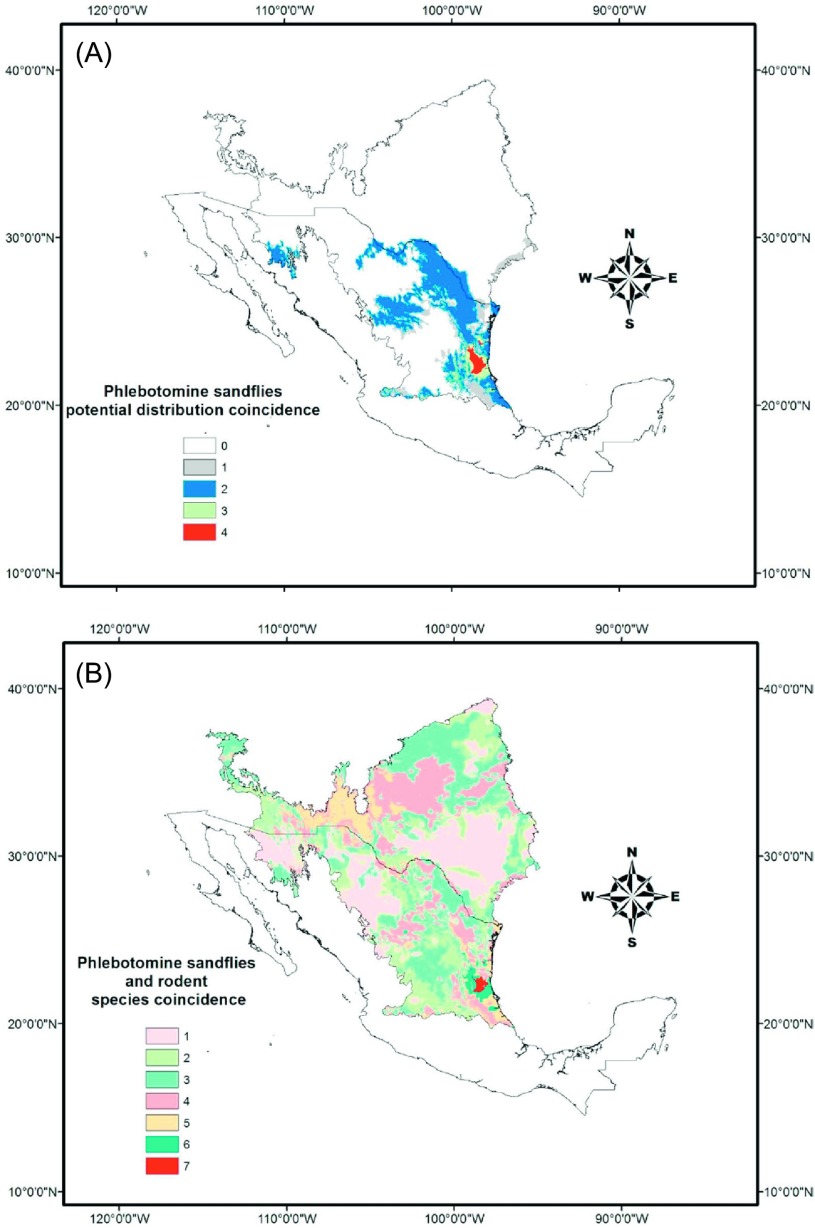
Potential distribution coincidence of four phlebotomine sandflies species (A), and *Leishmania*-infected rodent species (B). The red area shows the maximum coincidence at the southeast of the study area.

We made ENM models for only four *Lutzomyia* species, *Lutzomyia texana*, *Lu. shannoni*, *Lu. trinidadensis*, and *Lu. diabolica*. The sandfly ENMs constructed had an average AUC of 0.97 ± 0.004 and an accuracy that ranged from 0.89 to 0.97. Models showed a bias toward the Mexican territory due to the lack of georeferenced data from the US territory. Even this biased model showed that both *Lutzomyia texana* and *Lu. diabolica* can potentially be found in USA. The integrated map for four *Lutzomyia* predicted the presence of the four species to the limits of Veracruz and Chiapas, Mexico, and two *Lutzomyia* species on the Mexican-US border (Fig. 7A). The map integrating rodent and *Lutzomyia* species predicted south-Tamaulipas, Mexico as a place where infected-rodent species and *Lutzomyia* species could converge. The Mexican-US border is other area where at least one species of *Lutzomyia* and two of infected rodent coincide in their distributions (Fig. 7B).

## Discussion

Prior to this study, the information available on the distribution of sandfly species or disease records was basically presence of data and it was not possible to interpret the information on an ecological basis. This study represents the first contribution conducted in NEM that simultaneously documents both phlebotomine sandfly species as well as rodent species. As we standardized the same number and type of traps and the trapping efforts at each study site, it was possible to make collection data comparable between sites. Furthermore, this paper also represents the first ever record of *Leishmania* (*L.*) *mexicana* infection among wild-caught rodents in the NEM region.

Regarding phlebotomine sandfly fauna, our sampling effort detected 13 species, corresponding to 70% of the predicted species occurring in the NEM region according to Clench’s equation, which predicts that a total of 18 species should occur in the region. Many species are common to those reported in the USA [86, 87, 92].

Although we did not find *Leishmania* infection in female sandflies, that does not represent evidence of absence of parasites. It is possible that infection rates among female sandflies are relatively low; and perhaps a larger sample has to be analyzed. Natural infection in the sandfly *Lu. anthophora* has been reported to be 11.11% (3 out of 27 females) in collections made directly inside *Neotoma* nests in Bexar county, TX, USA [53]. In another study carried out in Bexar county, only one female *Lu. anthophora* (0.29%, 1 out of 347) was found infected with *Leishmania* parasites [50]. These quoted studies indicate that *Leishmania* infection may vary considerably even at the same study sites. The reasons for this variation are currently unknown. Similarly, natural infection rates of sandfly species from Southeastern Mexico [57, 59, 73] have also been found to vary between species, sites, and months of collection. For instance, infection rates reported by Pech-May et al. [57] varied from overall values of 5.4–66.7%; and for particular species, the infection rates were as follows: *Lu. cruciata* (25%; *n* = 1/4), *Lu. olmeca olmeca* (14.3–40%; *n* = 5/35; *n* = 15/35, respectively), *Lu. panamensis* (66.7%; *n* = 1/3), and *Lu. shannoni* (5.4–40.0%, *n* = (3/56); *n* = 8/20, respectively). Likewise, Sánchez-García et al. [73] also reported varied infection rates among *Lu. cruciata* (100%; *n* = 6/6), *Lu. olmeca olmeca* (0.2–20%), and *Lu. shannoni* (28.5%; *n* = 2/7). More recently, Pech-May et al. [59] reported a combined infection rate of 0.3% in Once de Mayo, Calakmul, Campeche. Infected flies were *Lu. olmeca olmeca* (0.3%; *n* = 1/342) and *Lu. shannoni* (0.2%, *n* = 1/412). For all the above reasons, we consider that to gain a better understanding of the natural infection rates, systematic and repetitive sampling needs to be undertaken in order to increase the chances of finding infected female sandflies. Despite the fact that we did not find natural *Leishmania* infection among the analyzed female sandflies, it is important to consider two interesting aspects: i) the presence of medically important species such *Lu. cruciata*, *Lu. diabolica, Lu. anthophora,* and *Lu. shannoni*; ii) *Leishmania* infection in wild mammals or humans occurs through the bite of an infected female sandfly. Thus, the infected rodents found in this study must have acquired an infection by some unknown sandfly vector. The role of vectors of *Leishmania* parasites by the above-mentioned species has been pointed out by several studies. For instance, *Lu. anthophora* is thought to be primarily an enzootic vector based on its close association with wood rats *Neotoma micropus* Baird [51]. Furthermore, *Lu. anthophora* has also been shown to transmit *Leishmania* parasites to experimental mice during blood feeding [20], and in Texas, *Leishmania* (*L*.) *mexicana* was isolated from wild-caught female *Lu. anthophora* [50]. As *Lu. anthophora* tends to be more associated with *Neotoma* spp. rats, it has been suggested by McHugh et al. [51] that the sandfly *Lu. diabolica* is a more likely vector in the light of its marked anthropophily. *Lu. diabolica* is a suspected vector in Texas and apart from the ecological data, there is experimental evidence that female *Lu. diabolica* are capable of maintaining and achieving full development of *Leishmania* (*L*.) *mexicana* parasites [45]. Furthermore, experimental transmission bioassays have also been conducted with *Lu. diabolica*, demonstrating the actual vector competence of females to transmit metacyclic promastigotes to hamsters [44]. With regard to the sandfly *Lu. cruciata*, there is growing evidence that this species could be an important vector of *Leishmania* spp. in southern Mexico, as this species has a large distribution area [26] and has been found naturally infected in several localities of Campeche [57, 59] and Quintana Roo [73]. Furthermore, this species is considered to be highly anthropophilic [4, 67], and it has been reported that *Lu. cruciata* is capable of transmitting *Leishmania* spp. parasites to hamsters under experimental conditions [90]. In the NEM region, *Lu. cruciata* is rarely found. However, to our knowledge, this species does occur in Texas and apparently, it is more commonly found in Florida [92]. Thus, the actual role of *Lu. cruciata* as a vector of *Leishmania* (*L*.) *mexicana* in NEM is yet to be determined in further studies. Another species with a large distribution area is *Lu. shannoni,* and it has been demonstrated that at least under laboratory conditions, female *Lu. shannoni* can harbor infectious stages of *Leishmania* (*L*.) *mexicana* [45]. In experimental bioassays, it has also been shown that infectious bites of laboratory-reared female *Lu. shannoni* can lead to cutaneous lesions in hamsters [44]. Canine infections in the USA [74] are thought to be transmitted by *Lu. shannoni*; however, no field data have been found to support the hypothesis put forward by Petersen [60, 61] and Petersen and Barr [62]. The role of the remaining species found in this study, as vectors of *Leishmania* parasites, remains an open question and although little evidence exists in the literature, the possibility that some of these species may actually transmit *Leishmania* parasites among other species of vertebrate hosts cannot be ruled out.

The alpha diversity indices we have used provide an ecological framework in which we can interpret the fact that the study site of Gómez Farías in the state of Tamaulipas had the highest effective number of species of phlebotomine sandflies. Results from the χ^2^ test (*r* × *c*) for association indicate that sandfly species are not evenly distributed over the study sites. As all sites varied in weather and/or vegetation types, however, it is not surprising that sandflies exhibited a heterogeneous pattern of distribution. Interestingly, results from beta diversity indices revealed that the similarity between sites was not associated with their proximity. For example, we found that two particular study sites (Santiago and Linares), that are geographically close to each other, did not share the same similarity in sandfly species. Other factors may affect the distribution of species and one possible factor could be the physical barrier of the mountains in the area. The presence of sandfly species at all study sites did not exhibit an aggregated pattern of co-occurrences and statistically, it was found that occurrences were random based on the C-score test. Of course, perhaps at the geographical scale of our study, species co-occurrences appear to be random, but if we consider a larger scale, it is possible that species co-occurrences could display an aggregated pattern.

For rodent species, it has been reported in several studies that, in Texas, *Leishmania* (*L*.) *mexicana* infection occurs in the wood rat *N*. *micropus* [29, 49, 66] and in *Neotoma floridana* (Ord) [52]; whereas, in Arizona, Kerr et al. [43] reported *Leishmania* (*L*.) *mexicana* infection in two *Neotoma albigula* Hartley. In the present investigation, we did not find any infection in *Neotoma* rats with all infected animals belonging to the genera *Sigmodon* and *Peromyscus*. All 16 *Neotoma micropus* rats collected and analyzed in this study were negative for the presence of *Leishmania* infection. Prevalence of infection of *N. micropus* in Texas ranges from 5.6% to 27% [42]. In mark-release-recapture studies carried out in Bexar county, TX, Raymond et al. [66] also reported that prevalence of infection among *N. micropus* rats varied from 3.8% to 26.7%. In our study, another interesting finding is that only *S. hispidus* was included in a list of 150 predicted mammal species with potential roles as hosts of *Leishmania* spp. [78]. In this list of potential reservoirs, *S. hispidus* was ranked number 27 with an epsilon value of 7.28; *P. eremicus, P. leucopus*, and *P. maniculatus* were not listed in this prediction list, and our findings would represent the first evidence of their putative role as reservoir hosts. A further risk model analysis of land cover variables and the co-occurrence of mammal species and sandfly species was conducted by González-Salazar et al. [27] indicating areas of low and high probability of transmission. According to these predictions, *Leishmania*-infected rodents found in this study overlap the areas of predicted high risk, which in turn indicates the power and validity of prediction models. In a more recent paper, Stephens et al. [79] expanded their model to the analysis of biotic networks among mammal and sandfly species. Therefore, the positive results of infection reported in this contribution fit those predictions well. At this stage, it is unclear why our results of infected rodents differ from those obtained in the Southern USA. One possibility is that studies in this region may perhaps have focused on *Neotoma* rats. Another possibility is that the southern USA in Texas bears more similarity with the northern counties of the Mexican states of Coahuila, Nuevo León, and Tamaulipas, and therefore it would be plausible that *Neotoma* spp. rats are more abundant in those areas. Our study sites in NEM were not really close to the border, so we do not know whether this factor plays a role. Alternatively, it is also probable that not many other species than *Neotoma* spp. have been screened for *Leishmania* infection in the USA. We analyzed tissues from all animals caught in the Sherman traps and did not focus our efforts on *Neotoma* spp. Interestingly, Kerr et al. [42] did not find *Leishmania*-positive individuals of *S*. *hispidus*, and suggested that the ecological association of *Neotoma* spp. rats and the sandfly vectors was one piece of evidence to support the hypothesis of the primary reservoir. Nevertheless, the most important point in our results is that they represent the first documented *Leishmania* (*L*.) *mexicana* infection in rodents in northern Mexico.

Over the last few years, there has been greater awareness of the disease in the state of Texas, with several authors pointing to an increase of known autochthonous cases of leishmaniasis there [11, 21, 48, 51, 91] in Oklahoma [11] and more recently, as far north as North Dakota [18]. In addition to the human cases reported in Texas, cutaneous and visceral leishmaniases have also been reported in dogs [19, 61, 74] and cats [14, 82]. It is still debatable as to whether or not there has been an expansion of the disease, as suggested by Wright et al. [91], or alternatively that the recent reported cases may actually be a consequence of human intrusion into areas of enzootic cycles [53]. A more recent study, published by Moo-Llanes et al. [56], predicted the future distributions (2020, 2050, and 2080) of 28 Central and North American sandfly species under the scenario of climate change. From this later study, it was predicted that most sandfly species would increase their distribution areas to the north, and thereby more people would be at risk of acquiring leishmaniasis. In contrast with the knowledge and awareness of the disease in the USA by public health authorities, in Mexico, such awareness is largely absent.

Currently, the magnitude of the epidemiological risk to the inhabitants of the NEM region from leishmaniasis is unknown. Although officially no recent cases have been reported in NEM, this does not mean that cases do not exist. Taking into account: i) that historically there have been cases of leishmaniasis in NEM; ii) the confirmed *L. mexicana* infection in putative reservoir hosts; and iii) that medically important sandfly species do occur in the area, there is substantial evidence to indicate that *Leishmania* parasites may be maintained in the wild in enzootic cycles, and perhaps the real threat of an outbreak is yet to be evaluated.

Related to this is the fact that four of the nine species collected have been considered as crop plagues. In fact, one of the *Leishmania*-positive rodents was caught in a sorghum crop field. The potential distribution models showed that practically the entire study area is covered by more than one rodent species. *Lutzomyia* models are biased due to the lack of georeferenced data in the whole of the study area mainly in the northernmost part (USA). This highlights the need for more studies focusing on documenting phlebotomine sandflies fauna in border area. However, geographically speaking, suitable habitats exist for convergence of biological elements of the *Leishmania* cycle. The ENMs suggest that *Leishmania* parasites could have spread from South Mexico to NEM and the Southeastern USA. Even though it has been documented that *Neotoma* species have a positive relationship with *Leishmania*, our findings indicated that other rodent species could be potential reservoirs as well. In a relatively recent publication, Wright et al. [91] documented the spread of leishmaniasis toward North Texas. ENMs of species such as those of the *Peromyscus* group and those of *Lu. texana* clearly indicated that this geographic range extension of the *Leishmania* parasite would been occurring with the help of rodent species that nowadays are not only a natural element in the regional fauna but are recognized as crop pests as well.

Recently, it has been proposed that leishmaniasis is one of the poverty-linked diseases shared by Mexico and the USA, with poor housing identified as a relevant factor [32, 33]. However, the ecology and biogeography of the disease surely play a fundamental role in its dynamics and associated risk. In this context, it is important to mention that potential reservoir hosts can also be pests of several crops, a situation predicted in our ecological niche models. A spread of crop pests would potentially be a contributing factor in the emergence of leishmaniasis in this area.

## Conflict of Interest

The authors declare that they have no competing interests.
